# Quantifying superspreading for COVID-19 using Poisson mixture distributions

**DOI:** 10.1038/s41598-021-93578-x

**Published:** 2021-07-08

**Authors:** Cécile Kremer, Andrea Torneri, Sien Boesmans, Hanne Meuwissen, Selina Verdonschot, Koen Vanden Driessche, Christian L. Althaus, Christel Faes, Niel Hens

**Affiliations:** 1 Interuniversity Institute for Biostatistics and statistical Bioinformatics, Data Science Institute, Hasselt University, Hasselt, Belgium; 2 Centre for Health Economics Research and Modelling Infectious Diseases, Vaccine and Infectious Disease Institute, University of Antwerp, Antwerp, Belgium; 3 Faculty of Sciences, Hasselt University, Hasselt, Belgium; 4 Division of Pulmonology, Department of Pediatrics, Antwerp University Hospital, Edegem, Belgium; 5 Radboud Center for Infectious Diseases, Radboud University Medical Center, Nijmegen, The Netherlands; 6 Institute of Social and Preventive Medicine, University of Bern, Bern, Switzerland

**Keywords:** Viral infection, Epidemiology

## Abstract

The number of secondary cases, i.e. the number of new infections generated by an infectious individual, is an important parameter for the control of infectious diseases. When individual variation in disease transmission is present, like for COVID-19, the distribution of the number of secondary cases is skewed and often modeled using a negative binomial distribution. However, this may not always be the best distribution to describe the underlying transmission process. We propose the use of three other offspring distributions to quantify heterogeneity in transmission, and we assess the possible bias in estimates of the mean and variance of this distribution when the data generating distribution is different from the one used for inference. We also analyze COVID-19 data from Hong Kong, India, and Rwanda, and quantify the proportion of cases responsible for 80% of transmission, $p_{80\%}$, while acknowledging the variation arising from the assumed offspring distribution. In a simulation study, we find that variance estimates may be biased when there is a substantial amount of heterogeneity, and that selection of the most accurate distribution from a set of distributions is important. In addition we find that the number of secondary cases for two of the three COVID-19 datasets is better described by a Poisson-lognormal distribution.

## Introduction

For any communicable disease, the basic reproduction number, $R_0$, denotes the average number of secondary cases a single infected individual generates in a completely susceptible population^1,2^. The basic reproduction number is considered to be of constant value among population members or specific population groups. However, for person-to-person transmitted infections, a complex combination of host, pathogen, and environmental factors defines the transmission potential of an infected individual, i.e. the number of other individuals a case infects during their infectious period^3,4^. It has been shown that, for a given $R_0$, both the probability that an epidemic will occur and the subsequent course of the epidemic are affected by individual variation in transmission^3^. Variation in disease transmission may lead to the existence of ‘superspreaders’ who infect substantially more individuals than others. When superspreading plays an important role during the epidemic, a relatively small proportion of infected cases will be responsible for most of the transmission, while many cases do not transmit the disease at all. Furthermore, when variation in disease transmission is present, large outbreaks can occur even if $R_0$ is less than one. To account for this heterogeneity, the individual number of secondary cases can be described by a random variable, whereas $R_0$ represents the expected value for an entire susceptible population.

The transmission potential of infected individuals can be seen as a combination of their biological infectiousness (i.e. viral shedding) and their contact behavior^5^. It is reasonable to assume that individuals with a higher viral shedding will be more likely to transmit the infection given a contact^6^. In addition, for a fixed level of viral shedding, infectious individuals with a higher contact rate will be more likely to generate secondary cases. Regarding SARS-CoV-2, significant individual variation in viral shedding has been reported^7^ and it has been argued that small aerosols exhaled during normal speech may serve as an important transmission route^8^. Vuorinen et al.^7^ investigated the possibility of SARS-CoV-2 transmission by inhalation of virus-containing aerosols, by examining a high-risk scenario where an infected individual coughs within a public indoor space. They found that there was an elevated risk of infection in case of lengthy exposure in a confined space with at least one infected individual. These results are in line with those from another study which has indicated that the virus may remain infectious as an aerosol for at least three hours^9^. Of course, not only individual characteristics such as viral shedding but also environmental characteristics such as insufficient ventilation contribute to the possibility of a superspreading event (SSE)^10^.

Lloyd-Smith et al.^3^ addressed heterogeneity in transmission by using the concept of an individual reproduction number as a random variable that represents the expected number of secondary cases caused by a particular infected individual. In that framework, SSEs are important realizations from the right-hand tail of the distribution of the individual reproduction number. Most studies investigating the amount of heterogeneity in disease transmission have assumed a Poisson process with rate given by the individual reproduction numbers, assumed to follow a Gamma distribution, resulting in a negative binomial offspring distribution^3,11^. In this way, heterogeneity has often been quantified using the *k* parameter, with *k* the negative binomial dispersion parameter. This has allowed comparison between studies, where lower values of *k* indicate increased heterogeneity in transmission, and thus possibly a larger amount of superspreading.

Based on this framework, a substantial amount of individual variation in the transmission of SARS-CoV-2 has been described, though large differences were found between different studies. Bi et al.^12^ used a negative binomial distribution to describe superspreading in the COVID-19 outbreak in Shenzhen, China, and found that about 9% of all cases were responsible for 80% of transmission. Riou and Althaus^13^ estimated the negative binomial dispersion parameter *k* to have a median of 0.54 (90% HDI 0.014–6.95), with simulations suggesting that very low values of overdispersion ($<0.1$) are less likely. Adam et al.^14^ estimated the overall mean number of secondary cases to be 0.58 (95%CI 0.45–0.72) with a dispersion parameter *k* of 0.43 (95%CI 0.29–0.67) in Hong Kong, indicating that 19% of cases were responsible for 80% of all local transmission. Similarly, Endo et al.^15^ have used a branching process model where the number of secondary cases was assumed to follow a negative binomial distribution. Assuming $R_0$ to be 2.5, they estimated the dispersion parameter *k* to have a median of 0.1 (95% CrI 0.05–0.2), resulting in 80% of secondary cases being caused by about 10% of infectious cases and implying that large transmission events should be prevented in order to contain epidemic spread. Laxminarayan et al.^16^ estimated the negative binomial dispersion parameter *k* to be 0.51 (95%CI: 0.49-0.52) using a large contact tracing dataset from two Indian states. Based on detailed contact tracing data from Hunan, China, Sun et al.^17^ found that 15% of cases were responsible for 80% of transmission, and a negative binomial dispersion parameter *k* of 0.3. Lau et al.^18^ found that superspreading was widespread across space and time, with an increasing presence towards later stages of the investigated outbreaks, highlighting the importance of maintaining social distance measures. They also found that about 2% of the most infectious cases were directly responsible for 20% of all infections.

It is well-recognized in statistical literature that the distribution underlying a data generating mechanism imposes a certain mean-to-variance relationship, which in practice may be severely violated^19^. Despite this, the use of other distributions in infectious disease modeling that may just as well account for variation in disease transmission has been rather limited. Some studies suggest that SSEs follow a power-law distribution with fat tails, such as the generalized Pareto distribution^20^. Brooks-Pollock et al.^21^ have used a negative binomial as well as a Poisson-lognormal distribution to model the distribution of cluster sizes for tuberculosis in the United Kingdom (UK) and the Netherlands. In this study, the Poisson-lognormal distribution provided a better fit to the UK data, indicating the importance of comparing different assumptions about the underlying distribution when variation in disease transmission is present.

To our knowledge, there are no studies that have explicitly investigated the possible bias in using the negative binomial distribution as an approximation to the underlying transmission process. We argue that it is important to compare different distributional assumptions since different distributions could portray different tail behaviour, and hence capture SSEs differently. In this work we explore the use of other Poisson mixture distributions for inference of the offspring mean and the amount of heterogeneity in disease transmission. We focus on the three-parameter generalized Gamma distribution for the individual reproduction number, because of its flexibility and the fact that it has as special cases the Gamma, Weibull, and lognormal distribution^22^. First, we carry out a simulation study to investigate the potential bias in the estimation of the offspring mean and its variance when the distribution that is fit to the data does not correspond to the actual data generating distribution. Next, we use the proposed distributions to (re-)analyze several COVID-19 datasets from Rwanda, India, and Hong Kong, and investigate the impact of the considered offspring distribution on the estimation of the proportion of cases that is responsible for 80% of transmission, $p_{80\%}$. These countries were chosen because contact tracing data were available to construct empirical offspring distributions.

## Results

### Simulation study

We consider the Poisson-generalized Gamma (POGG) and three submodels for the offspring distributions: negative binomial (NB), Poisson-lognormal (POLN), and Poisson-Weibull (POWB). See Methods for a description of these Poisson mixture distributions and how the simulation study was performed. In general we find that when overdispersion increases, estimates tend to become more biased when the considered offspring distribution does not correspond to the data generating distribution (Suppl. Tables S2 & S3). This is especially the case when considering estimates of the standard deviation. As overdispersion increases, the true distribution is more often considered as the best fit based on AIC. In particular, when the data generating mechanism deviates from the NB model, assuming the NB model will often lead to an underestimation of the standard deviation. Where results are missing, no estimates could be obtained.

#### Expected versus realized proportions of transmission

**Figure 1:**
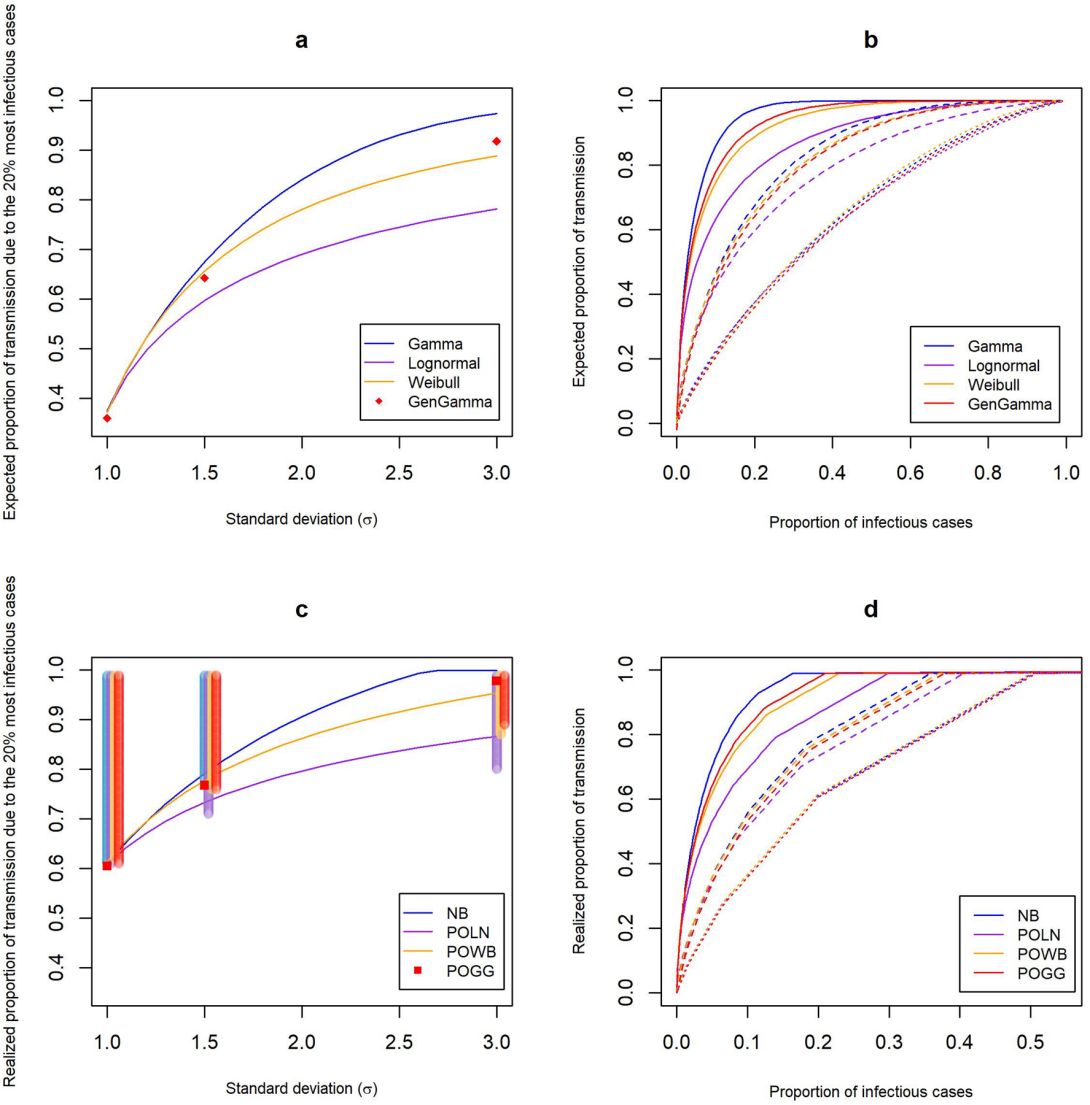
The top panel shows the expected proportion of all transmission that is **(a)** due to the 20% most infectious cases for different levels of overdispersion and different distributions, with the offspring mean *R* fixed at 0.8; and **(b)** due to a given proportion of infectious cases, where cases are ranked by their transmission potential, for $\sigma = 1$ (dotted), $\sigma = 1.5$ (dashed), $\sigma = 3$ (full), and the different distributions, with *R* fixed at 0.8. The lower panel shows the realized proportion of all transmission that is **(c)** due to the 20% most infectious cases, shaded vertical bars show the range surrounding the proportions at $\sigma \in \{1,1.5,3\}$ (see Supplementary Methods); and **(d)** due to a given proportion of infectious cases.

Based on the estimated mean and variance of the considered offspring distribution, we can obtain estimates for the proportion of cases responsible for a certain amount of transmission. There are two different approaches for obtaining these proportions (see Methods), where one is based on the distribution of the individual reproduction number^3^, and the other is based on the complete offspring distribution^15^. Here we show how the different offspring distributions can affect these proportions. Figure 1a shows the expected proportion of transmission due to the 20% most infectious cases for the varying levels of heterogeneity used in the simulation study, for the different offspring distributions. For all distributions, this proportion increases with an increasing amount of heterogeneity (i.e. higher $\sigma$, which for the negative binomial results in lower *k*). Thus, less heterogeneity leads to a smaller proportion of transmission being attributed to the 20% most infectious cases. In case of high overdispersion, there is a substantial difference in the expected proportions between the distributions. Since for the Poisson-generalized Gamma distribution it is not possible to specify the parameters from a given mean and variance, we only estimated these proportions at the specified settings used in the simulation study, hence these are represented as dots instead of lines. Figure 1b shows the expected proportion of transmission due to a given proportion of infectious cases for the different levels of overdispersion. It can again be seen that the difference between these estimates across the different distributions increases when overdispersion increases (i.e. higher $\sigma$) and likewise there is a substantial difference in terms of the expected proportions of transmission. When taking into account the additional variation coming from the Poisson process, the same increase in the proportion of transmission due to the 20% most infectious cases is seen, again with substantial differences between the distributions when overdispersion increases (Fig. 1c,d). These results also hold when $R>1$ (Suppl. Fig. S2). The vertical bars in Fig. 1c represent the uncertainty in the proportion of transmission due to the discrete nature of the offspring distribution and should be interpreted as the range of transmission that will be due to the 20% most infectious cases. When taking into account this range surrounding these point estimates, a substantial difference is seen between the Poisson-lognormal and the other distributions for higher levels of overdispersion.

### Application to COVID-19 data

**Figure 2:**
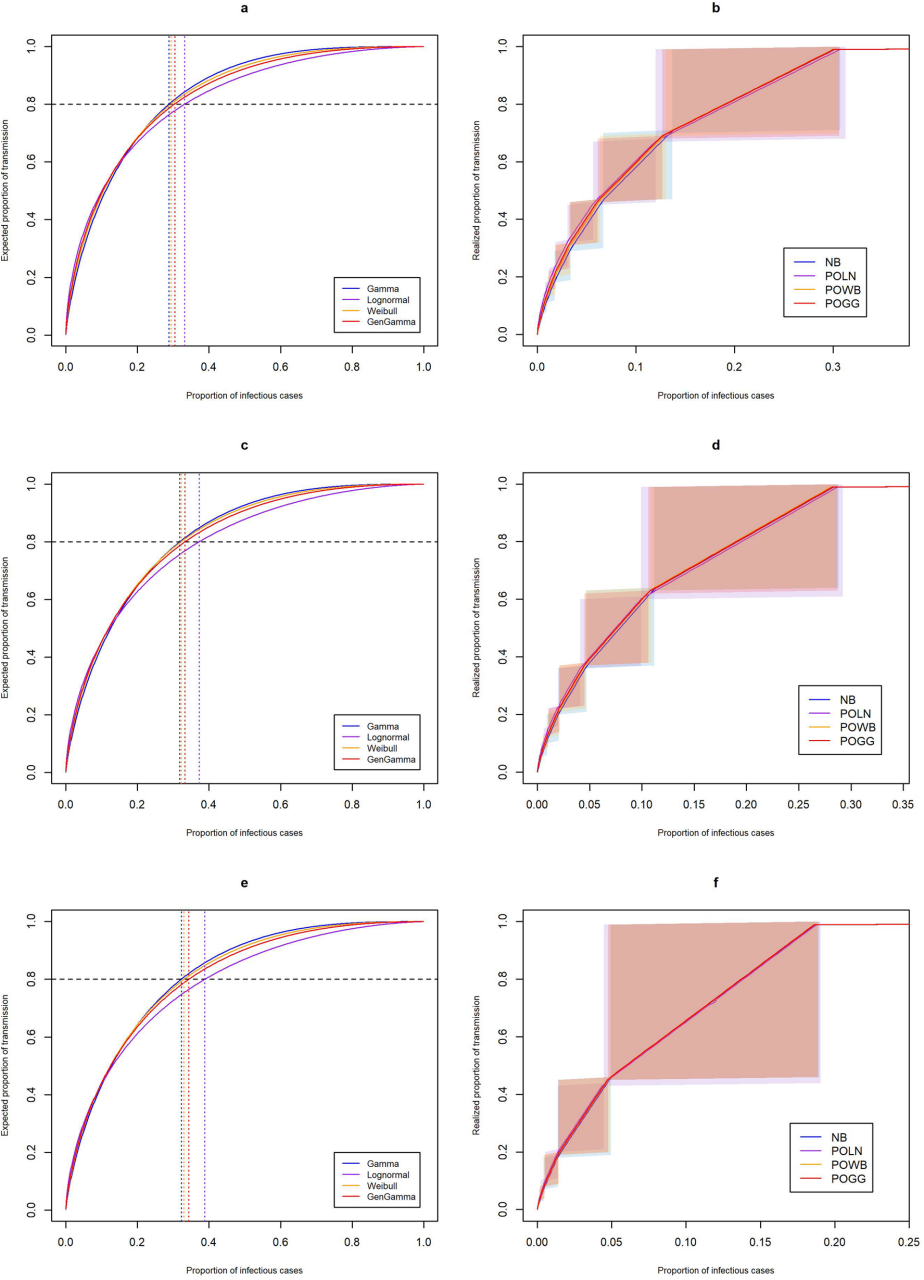
Proportion of most infectious cases responsible for a certain proportion of transmission, based on estimates from **(a,b)** Hong Kong, **(c,d)** India, and **(e,f)** Rwanda. Proportions are obtained based on the distribution of the individual reproduction number (left), and based on the complete offspring distribution (right). The shaded areas in the right panels represent the range surrounding specific proportions when considering the discrete nature of the realized offspring distributions (see Supplementary Methods).

Table 1 shows estimates of the offspring mean *R* and standard deviation $\sigma$ obtained by fitting the different offspring distributions to each COVID-19 dataset. In terms of AIC and the corresponding Akaike weights, the data from Hong Kong and India are best described by a Poisson-lognormal distribution. Supplementary Figs. S4, S6, and S8 show the fit of the different distributions to the observed offspring distribution. It can be seen that for the data from Hong Kong and India the negative binomial, Poisson-Weibull, and Poisson-generalized Gamma distributions do not adequately capture the proportion of cases that generate only one secondary case, while this is captured well by the Poisson-lognormal distribution. For the data from Rwanda this is less evident. Goodness-of-fit plots are shown in Suppl. Figs. S5, S7, and S9. For each of the three datasets (Table 2), $p_{80\%}$ is estimated to be substantially higher for the Poisson-lognormal than for the other distributions when based on the distribution of the individual reproduction number (equation (1), see Methods), and slightly higher when taking into account additional random variation from the Poisson process (equation (2), see Methods). Figure 2a,c,e show the expected proportions of transmission due to a certain proportion of cases for each distribution and each dataset, while Fig. 2b,d,e show the realized proportions. For Hong Kong, we estimate that roughly 12-31% of cases are responsible for 68-100% of all transmission based on the Poisson-lognormal distribution (Fig. 2b), while based on the negative binomial distribution, we estimate that roughly 14-31% of cases are responsible for 71-100% of all transmission. For India, we estimate that roughly 10-29% of cases are responsible for 61-100% of all transmission based on the Poisson-lognormal distribution (Fig. 2d), while based on the negative binomial distribution, we estimate that roughly 11-29% of cases are responsible for 64-100% of all transmission. For Rwanda, we estimate that roughly 5-19% of cases are responsible for 44-100% of all transmission based on the Poisson-lognormal distribution (Fig. 2f), while based on the negative binomial distribution, we estimate that roughly 5-19% of cases are responsible for 46-100% of transmission.

**Table 1:** Estimates of the offspring mean *R* and its standard deviation ($\sigma$), in addition to the coefficient of variation (*CV*), using the different mixture distributions, and their AIC value and corresponding Akaike weights ($w_i$), for three COVID-19 datasets.

Dataset	Distribution	*R* (95%CI)	$\sigma$ (95%CI)	*CV* (95%CI)	AIC	$w_i$
Hong Kong^14^	NB	0.583 (0.448–0.718)	1.175 (0.944–1.490)	2.016 (1.816–2.395)	593.925	0.078
	POLN	0.587 (0.456–0.779)	1.413 (0.969–2.442)	2.409 (1.869–3.426)	590.009	0.551
	POWB	0.580 (0.445–0.745)	1.218 (0.970–1.734)	2.101 (1.841–2.560)	591.747	0.231
	POGG	0.580 (0.3789–0.724)	1.258 (0.923–1.550)	2.171 (2.059–2.466)	592.738	0.141
India^16^	NB	0.484 (0.480–0.494)	0.973 (0.962–0.985)	2.009 (1.994–2.024)	163974.5	0.000
	POLN	0.484 (0.477–0.491)	1.077 (1.055–1.101)	2.226 (2.195–2.258)	162980.6	0.000
	POWB	0.483 (0.476–0.489)	0.997 (0.984–1.011)	2.067 (2.049–2.084)	163530.8	1.000
	POGG	0.484 (0.477–0.490)	1.012 (1.000–1.024)	2.094 (2.086–2.102)	163286.5	0.000
Rwanda	NB	0.259 (0.216–0.302)	0.623 (0.547–0.731)	2.406 (2.223–2.743)	1015.261	0.157
	POLN	0.260 (0.219–0.311)	0.657 (0.560–0.820)	2.528 (2.267–2.892)	1013.073	0.468
	POWB	0.259 (0.217–0.311)	0.631 (0.557–0.783)	2.436 (2.225–2.750)	1014.350	0.247
	POGG	0.259 (0.216–0.301)	0.634 (0.561–0.706)	2.451 (2.340–2.603)	1015.667	0.128

**Table 2:** Estimates of the proportion of cases responsible for 80% of transmission ($p_{80\%}$, following Eqs. (1) and (2) using the different mixture distributions, for three COVID-19 datasets. Estimates based on the negative binomial distribution correspond to those reported in the literature for the two published datasets.

Dataset	Distribution	$p_{80\%}$ (95%CI)	
		Eq. (1)	Eq. (2)
Hong Kong^14^	NB	0.288 (0.208–0.345)	0.191 (0.145–0.223)
	POLN	0.332 (0.236–0.438)	0.195 (0.153–0.242)
	POWB	0.294 (0.223–0.358)	0.189 (0.148–0.221)
	POGG	0.303 (0.279–0.325)	0.190 (0.145–0.203)
India^16^	NB	0.319 (0.314–0.324)	0.191 (0.189–0.194)
	POLN	0.373 (0.367–0.379)	0.195 (0.193–0.199)
	POWB	0.322 (0.318–0.327)	0.189 (0.187–0.191)
	POGG	0.333 (0.332–0.335)	0.191 (0.189–0.192)
Rwanda	NB	0.323 (0.223–0.390)	0.138 (0.114–0.157)
	POLN	0.389 (0.318–0.459)	0.139 (0.120–0.157)
	POWB	0.331 (0.241–0.394)	0.137 (0.117–0.157)
	POGG	0.344 (0.337–0.350)	0.137 (0.122–0.151)

## Discussion

Since most studies that aim to quantify variation in disease transmission have assumed the offspring distribution to follow a negative binomial, we investigated the impact of incorrectly assuming this distribution as an approximation to the underlying transmission process. Results from our simulation study show that when overdispersion increases, estimates of the offspring mean and especially its variance can become quite biased when making incorrect assumptions about the underlying data generating distribution. This conclusion remains valid when using simulated offspring distributions for only 1000 cases (see Supplementary Table S3). When no variation in transmission is present, all distributions performed equally well, although there was a slightly increased bias in variance estimates when using the Poisson-Weibull or Poisson-generalized Gamma distribution. We have (re-)analyzed three COVID-19 datasets and for two of these datasets (i.e. Hong Kong and India) the Poisson-lognormal distribution gave the best fit to the observed data in terms of AIC, which was confirmed by the Akaike weights assigning the highest probability to this model. This resulted in considerable differences in terms of the expected $p_{80\%}$ compared to when using a negative binomial distribution, when these proportions were based on the distribution of the individual reproduction number^3^. For India, $p_{80\%}$ was estimated to be significantly higher when based on the Poisson-lognormal compared to the other distributions. When accounting for the additional variation introduced by the Poisson process, the differences in these proportions of cases responsible became negligible. For example, for the Hong Kong data the point estimate when using the Poisson-lognormal was 19.5%, compared to the previously reported 19.1% when using the negative binomial^14^. When accounting for the discrete nature of the offspring distribution, estimated ranges for these proportions were mostly overlapping for the different distributions, albeit a bit lower for the Poisson-lognormal distribution. Although most studies report $p_{80\%}$, the right-sided panels in Fig. 2 indicate that depending on the proportion of transmission one is interested in, there might be a more substantial difference between the distributions. This implies that different distributions have different tail properties, underlining the importance of investigating which distribution best describes the data at hand. In addition we found that the difference between the distributions increases with an increasing level of overdispersion (Fig. 1). We do not suggest to always use the Poisson-lognormal distribution instead of a negative binomial, but rather recommend to compare different distributions and select the most accurate one, i.e. the best fitting one, as the true underlying process is always unkown. Our analysis of the Hong Kong data shows that model selection is important even when the sample size is relatively small.

Our analyses indicated that the negative binomial distribution often does not adequately capture the proportion of cases that generate only one secondary case, thereby possibly overestimating the importance of superspreading events. This overestimation was observed when the proportion of cases responsible was obtained based on the distribution for the underlying individual reproduction number. When accounting for the Poisson process, superspreading was found to be only slightly more important when using a negative binomial distribution to describe the data, compared to a Poisson-lognormal distribution. A negative binomial distribution enables easy comparison between different studies through its dispersion parameter *k*^3^. However, this should not be a reason to only use negative binomial offspring distributions without exploring other alternatives. In fact, estimates of *k* become meaningless when the negative binomial does not accurately describe the underlying distribution. The results from different studies can also be compared by their estimated $p_{80\%}$, which is often reported as well and can be obtained for any distribution that best describes the data. In addition, $p_{80\%}$ has a more intuitive interpretation than the negative binomial dispersion parameter *k*. It should be noted that there are different approaches for obtaining these proportions, and care should be taken when comparing these results between studies because of the difference in interpretation. Lloyd-Smith et al.^3^ assume SSEs to be realizations from the right-hand tail of the distribution of the individual reproduction number, hence their approach is based on this continuous distribution and only depends on the level of overdispersion (Suppl. Fig. S1a). In contrast, Endo et al.^15^ have based these estimates on the complete offspring distribution, taking into account additional variation arising from the discrete Poisson process. In this way, the second approach accounts for more heterogeneity. However, if the effective contact process in reality is not a Poisson process, this approach may result in biased estimates. We also indicate that these proportions based on the negative binomial or another discrete distribution should be expressed as a range instead of a fixed number, which has not been accounted for in previous studies.

In this study we have considered the three-parameter Poisson-generalized Gamma distribution, which has as special cases the Poisson-lognormal, Poisson-Weibull, and negative binomial distribution. Although the Poisson-generalized Gamma distribution has the advantage of being very flexible due to the additional parameter, the disadvantage is that because of this added complexity the estimation is computationally more extensive, especially for large datasets. Furthermore, parameter estimation can be difficult because different parameter sets can give rise to the same density function. In general, parameter estimation becomes more difficult when the amount of overdispersion is high and incorrect assumptions about the underlying data generating distribution are made. For that reason we were not able to fit the Poisson-Weibull and Poisson-lognormal distributions in some scenarios of our simulation study. This occurred when the data were highly overdispersed ($k<$ 0.1), which is less likely to be encountered in practice^13^.

Inference of the amount of heterogeneity in transmission is paramount for identifying a disease’s potential of superspreading. Correctly quantifying this heterogeneity is important because it affects estimates of other epidemiological parameters, modulates the degree of unpredictability of an epidemic, and needs to be taken into account when modeling disease control and planning control strategies^23^. When there is evidence of substantial superspreading, control measures should focus on limiting the potential for SSEs to occur by restricting large events and avoiding crowding in other public spaces. Typically, when control measures are taken, one aims to prevent transmission from those cases expected to have a high individual reproduction number, without knowing whether they will actually realize these secondary cases. Control measures thus act on the individual reproduction number and the expected transmissions, whereas their effect will be observed at the level of realized transmissions. In general, for a given proportion of individuals that are ‘controlled’, greater targeting of individuals with a higher expected number of secondary cases (e.g. those individuals that are expected to have a lot of contacts) will result in a lower effective *R* and higher extinction probability^3^. When $p_{80\%}$ is low, this implies that the majority of infected individuals do not transmit the virus and thus the epidemic cannot be sustained if superspreading events are prevented by e.g. restricting large gatherings. On the other hand, for higher $p_{80\%}$, more individuals will contribute to transmission and additional control measures that focus on regular contacts (e.g. work, school, family) become more important^24^. Also, because of the increased speed at which the epidemic spreads when SSEs are present, heterogeneity could lower doubling times^25^.

Detailed contact tracing data, including information about the time of data collection, are needed to obtain empirical offspring distributions, but these are often not available. Even if available, most of these datasets will be of limited sample size. In general, small samples might be less likely to include values from the right-hand tail of a distribution. Outbreak data obtained from contact tracing however tend to be biased toward including these right-hand tail observations because outbreaks that are detected are often those that go on for a few generations, tending to include a highly infectious individual among the early generations, and in this way counteracting the small-sample bias that would occur when these observations are lacking^26^. Although it has been shown that there is minimal risk of overestimating the negative binomial dispersion parameter *k* when using MLE with small samples^26^, we cannot be certain this also applies to other Poisson mixture distributions. However, we believe that for the purpose of our study MLE, and consequently AIC for model comparison, is a valid approach. When possible, temporal changes in the offspring distribution should be accounted for, especially when the dataset is large and collected over multiple time periods. This work should be extended such that the considered distributions can be used to infer the offspring mean *R* and its overdispersion from final size data^11^, which are often more readily available. In a recent study by Brooks-Pollock et al.^21^ it was found that assuming a branching process with a negative binomial distribution of secondary cases systematically underestimated the frequency of large tuberculosis clusters, while a Poisson-lognormal model was able to capture the entire distribution.

Rock et al.^27^ have mentioned the distinction between ‘super-spreaders’ and ‘super-shedders’, who are both responsible for an above average number of secondary cases but for different reasons. In a meta-regression analysis, Chen et al.^28^ investigated the relationship between the dispersion parameter *k* and respiratory viral load (rVL). They found that heterogeneity in rVL facilitates variation in individual infectiousness and hence may be associated with overdispersion in the number of secondary cases. Another recent study has shown that highly heterogeneous contact behavior is required to produce extreme superspreading^29^. Future work should thus aim to disentangle heterogeneity coming from variation in contact rates versus heterogeneity coming from variation in viral shedding. Furthermore, assuming a homogeneous Poisson process for effective contacts is likely a simplification of the real contact process, hence the use of other distributions to describe the contact process should also be considered. In a recent study, Wong and Collins^20^ have suggested that the offspring distribution for SARS-CoV-2 is fat-tailed, and that this tail part of the distribution is consistent with a generalized Pareto distribution. For completeness, we have fit discrete Pareto offspring distributions to the three datasets used in this study, but did not find evidence that this provided a better description of the complete offspring distribution (see Supplementary Table S1 and Supplementary Fig. S3). Overall, the results of the present study suggest that, whenever possible, several distributions should be compared in terms of their fit to the observed data before making inferences on the amount of heterogeneity because resulting conclusions on the importance of superspreading may be different.

## Methods

### Poisson mixture distributions

Differences in infectious disease transmission among individuals can arise either from differences in infectiousness or from differences in susceptibility, and can be interpreted in terms of the underlying contact and infection processes^30^. An effective contact is a contact that can lead to transmission, whereas an infectious contact occurs when an effective contact is realized between an infectious and susceptible individual. Effective contacts can be described using a Poisson counting process. Overdispersion occurs when the variance of a distribution exceeds its mean, which is not accounted for by a Poisson distribution with constant rate. Instead if the rate itself is a random variable drawn from a particular distribution, this overdispersion can be captured. When there is high overdispersion, a small proportion of infected individuals will be responsible for the majority of transmission. Let *Y* denote the effective contact process that follows a Poisson distribution, $Y \sim Po(\nu )$, where $\nu$ represents the individual reproduction number that allows for heterogeneity in transmission. The effective contact process *Y* is then described by a Poisson mixture distribution. In this work we focus on the three-parameter generalized Gamma distribution for $\nu$, because of its flexibility and the fact that it has as special cases the Gamma, Weibull, and lognormal distribution^22^. Table 3 shows the resulting Poisson mixture distributions, each with mean *R* and variance $\sigma ^2$. More details can be found in Supplementary Methods.

**Table 3:** Different mixture distributions, assuming a Poisson distribution for the effective contact process.

Distribution for $\nu$	Offspring distribution	*R*	$\sigma ^2$
$\nu \sim$ Ga($\alpha$,$\beta$)	$Y \sim$ NB$(\mu ,k)$	$\mu = \frac{\alpha }{\beta }$	$\mu (1+\frac{\mu }{k}) = \frac{\alpha }{\beta }(1+\frac{1}{\beta })$
$\nu \sim$ LogN($\mu _{log},\sigma _{log}$)	$Y \sim$ PoLN$(\mu _{log},\sigma _{log})$	$e^{\mu _{log}+\frac{\sigma ^2_{log}}{2}}$	$e^{\mu _{log}+\frac{\sigma ^2_{log}}{2}} + \big [ (e^{\sigma ^2_{log}} -1) e^{2\mu _{log}+\sigma ^2_{log}} \big ]$
$\nu \sim$ Weibull(*p*,*l*)	$Y \sim$ PoWB(*p*, *l*)	$l \Gamma (1+\frac{1}{p})$	$l\Gamma (1+\frac{1}{p}) + l^2 \big [ \Gamma (1+\frac{2}{p}) - \big (\Gamma (1+\frac{1}{p})\big )^2 \big ]$
$\nu \sim$ GG(*a*,*d*,*p*)	$Y \sim$ PoGG(*a*, *d*, *p*)	$a \frac{\Gamma (\frac{d+1}{p})}{\Gamma (\frac{d}{p})}$	$a\frac{\Gamma (\frac{d+1}{p})}{\Gamma (\frac{d}{p})} + a^2 \Big [\frac{\Gamma (\frac{d+2}{p})}{\Gamma (\frac{d}{p})} - \Big (\frac{\Gamma (\frac{d+1}{p})}{\Gamma (\frac{d}{p})} \Big ) ^2 \Big ]$

#### Simulation study

To assess the possible bias in estimates of the reproduction number *R* and its overdispersion, which are based on assuming a certain offspring distribution for the underlying transmission process, we investigate the influence of the choice of distribution on the corresponding estimates. For this purpose, we will use the standard deviation (SD) as a measure for quantifying the degree of overdispersion (Suppl. Fig. S10). Using each of the mixtures in Table 3 we generate 1000 datasets containing the distribution of secondary cases for 10,000 individuals, to avoid small-sample bias. We set the mean number of secondary cases (i.e. the offspring mean) to 0.8 to represent local epidemic outbreaks with control measures in place, and vary the standard deviation $\sigma \in \{1, 1.5, 3\}$ corresponding to different levels of overdispersion (negative binomial $k \in \{3.2, 0.44, 0.08\}$). We also consider a scenario without heterogeneity where the data are generated from a Poisson distribution with variance equal to the mean, $R = \sigma ^2 = 0.8$. We then estimate the parameters of the mixture distributions for each simulated dataset ($i=1,\dots ,1000$) using maximum likelihood estimation (MLE) and obtain the estimated mean $\hat{R_i}$ and standard deviation $\hat{\sigma _i}$ of the offspring distribution. For each distribution we calculate what we refer to as bias in the estimates as $\bar{{\hat{x}}} - x$, where *x* is the true value of the parameter of interest and $\bar{{\hat{x}}}$ is the sample mean. Following Burton et al.^31^, a bias larger than |0.5$SE({\hat{x}})$| is substantial, where $SE({\hat{x}})$ is the empirical standard error of the estimate ${\hat{x}}$ across all simulated datasets (i.e. the between-sample variability). We also obtain the bias as a percentage of the $SE({\hat{x}})$, which ideally would be smaller than 40% in either direction^32^. Further, we calculate the mean squared error (MSE) as a measure of overall accuracy by taking into account the bias as well as the variability in the estimates. For example, a more flexible model such as the Poisson-generalized Gamma distribution is expected to have lower bias, but as a consequence of its complexity the variability is expected to be higher^33^.

#### Expected versus realized proportions of transmission

After estimating the mean ${\hat{R}}$ and variance ${\hat{\sigma }}^2$ of the considered mixture distribution, we can obtain the proportion of cases responsible for a given proportion of transmission. Following Lloyd-Smith et al.^3^, the parameters ${\hat{R}}$ and $\sigma ^2 = {\hat{\sigma }}^2 - {\hat{R}}$ specify the probability density function (pdf) $f_\nu (x)$ and cumulative distribution function (cdf) $F_\nu (x)$ of the distribution describing the individual reproduction number $\nu$. The cdf for disease transmission is defined by

1 $$\begin{aligned} F_{trans}(x) = \frac{1}{{\hat{R}}} \int _0^x x f_\nu (x) dx \end{aligned}$$

and denotes the expected proportion of transmission due to infectious cases with $\nu < x$, while $1-F_{trans}(x)$ denotes the expected proportion of transmission due to those cases with $\nu > x$. If *p* is the proportion of transmission for which we want to know the expected proportion of cases responsible, *a*, we first need to find *x* such that $1 - F_{trans}(x) = p$. The value *x* then denotes the threshold value of the reproduction number for which $1 - F_{trans}(x)$ is the expected proportion of transmission *p* due to cases with $\nu > x$. We can then obtain the expected proportion of cases which have their reproduction number $\nu > x$ as $P(X>x) = 1-P(X\le x) = 1-F_\nu (x)$. This is the expected proportion of infectious cases *a* that is responsible for a proportion *p* of all transmission. Note that in case of a homogeneous Poisson process the relation between *a* and *p* will be linear (Supplementary Fig. S1b) because the variance of the mixing distribution will be zero.

If we want to take into account the additional variation coming from the Poisson process, we need to extend the method above for use with the Poisson mixtures (i.e. the offspring distributions). Endo et al.^15^ have done this for the negative binomial distribution and we extend this for the other mixtures in the following way. The cdf for disease transmission is now defined by

2 $$\begin{aligned} F_{trans}(x) = \frac{1}{{\hat{R}}} \int _0^x \lfloor x \rfloor f(\lfloor x \rfloor ) dx \end{aligned}$$

where $f(\lfloor x \rfloor )$ is the density function of the mixture distribution evaluated at the integer part of *x*. $F_{trans}(x)$ now denotes the proportion of transmission that is due to cases that have their number of secondary cases $r < x$. Again we first need to find *x* such that $1-F_{trans}(x) = p$, where *x* then denotes the threshold value of the reproduction number for which $1-F_{trans}(x)$ is the proportion of transmission *p* due to cases with $r \ge x$. The proportion of cases that have $r < x$ is defined as

3 $$\begin{aligned} F(x-1) = \int _0^x f(\lfloor x \rfloor ) dx. \end{aligned}$$

The proportion of cases that have their number of secondary cases $r \ge x$ is then $P(X \ge x) = P(X > x-1) = 1 - F(x-1)$. This is now the proportion of cases *a* that is responsible for a proportion *p* of all transmission. However, as this is a continuous approximation of a discrete distribution, we should account for “uncertainty” in these point estimates of *a* and *p* introduced by the fact that it is unlikely that there exists an integer *x* for which $1-F_{trans}(x)$ exactly equals *p*. To do this, we use a discrete version of the method proposed by Lloyd-Smith et al.^3^ (see details in Supplementary Methods). We then obtain a range for the proportion of cases *a* responsible for a certain proportion of transmission *p*, which is then also expressed as a range.

More details on the difference between these two approaches can be found in Supplementary Methods. Essentially, Lloyd-Smith et al.^3^ estimate the *expected* proportion of cases responsible, whereas Endo et al.^15^ estimate the *realized* proportion. We investigate the impact of the assumed offspring distribution on estimates of the proportion of infectious cases responsible for a certain amount of transmission.

### Application to COVID-19 data

Using MLE, we fit the different Poisson mixture distributions to three datasets containing the distribution of secondary cases for COVID-19. From the estimated parameters we calculate the mean *R* and standard deviation $\sigma$ of the offspring distribution, and obtain their 95% confidence intervals (CI) by sampling 100,000 values from a multivariate normal distribution for the parameters of the offspring distribution. We compare the models in terms of AIC (Akaike information criterion^34^, which indicates goodness-of-fit for a given model while penalizing model complexity), with lower values indicating a better description of the data, and goodness-of-fit based on observed vs. expected distribution of secondary cases. We further quantify model selection uncertainty using Akaike weights $w_i$. These weights can be interpreted as the probability that model *i* is the best model for the data, conditional on the full set of candidate models considered^35^. We also investigate the impact of the different distributions on the inference of $p_{80\%}$. Confidence intervals for $p_{80\%}$ are obtained by sampling 1000 values from a multivariate normal distribution for the parameters of the offspring distribution. We use two publicly available datasets, one containing the offspring distribution for 290 cases in Hong Kong^14^, and one containing the offspring distribution for 84,965 cases in India^16^. Hong Kong had successfully limited its number of confirmed cases during the early stages of the pandemic^36^, but has since then seen several resurgences^37^. Although India responded rapidly by imposing a strict lockdown, it is now one of the worst-affected countries in Asia, and has the second highest number of infections worldwide^38^. The third dataset contains the offspring distribution for 795 cases in Rwanda (personal communication), which had a cumulative number of COVID-19 cases of 6.4 per 10,000 population on December 31, 2020. Rwanda has used a multi-sectoral approach to contact tracing by involving community health worker teams and local government authorities^39^ These datasets were chosen because of availability at the time of our analyses. Other country-specific studies, including France, China, Canada, and Italy, on COVID-19 have been reported in literature, but at the time of analysis those contact tracing data were not publicly available^40,41^. Validity of the MLE/AIC approach was confirmed by a positive-definite Fisher information matrix for each model under each dataset, indicating no singularity issues.

## Supplementary Information

Supplementary Information.

## Data Availability

The data from Hong Kong and India used in this study are publicly available. The empirical offspring distribution from Rwanda and the code to generate data as used in the simulation study are available on GitHub, as well as all relevant R code to perform the analyses (https://github.com/cecilekremer/PoiMixtSS).
